# Light Field Imaging Based Accurate Image Specular Highlight Removal

**DOI:** 10.1371/journal.pone.0156173

**Published:** 2016-06-02

**Authors:** Haoqian Wang, Chenxue Xu, Xingzheng Wang, Yongbing Zhang, Bo Peng

**Affiliations:** 1 Shenzhen Key Laboratory of Broadband Network & Multimedia, Graduate School at Shenzhen, Tsinghua University, Shenzhen, China; 2 Department of Software Engineering, Southwest Jiaotong University, Chengdu, China; Huazhong University of Science and Technology, CHINA

## Abstract

Specular reflection removal is indispensable to many computer vision tasks. However, most existing methods fail or degrade in complex real scenarios for their individual drawbacks. Benefiting from the light field imaging technology, this paper proposes a novel and accurate approach to remove specularity and improve image quality. We first capture images with specularity by the light field camera (Lytro ILLUM). After accurately estimating the image depth, a simple and concise threshold strategy is adopted to cluster the specular pixels into “unsaturated” and “saturated” category. Finally, a color variance analysis of multiple views and a local color refinement are individually conducted on the two categories to recover diffuse color information. Experimental evaluation by comparison with existed methods based on our light field dataset together with Stanford light field archive verifies the effectiveness of our proposed algorithm.

## Introduction

Image specular reflection has long been problematic in computer vision tasks [1]. They appear as surface features, but in fact they are artifacts caused by illumination changes from different viewing angles [2]. Most algorithms in computer vision such as segmentation [3] (which typically assumes the intensity changes uniformly or smoothly across a surface), or stereo matching [4], recognition [5–9], image analysis [10–14]and tracking [15] (they attempt to match images taken from various conditions, i.e., viewing angle, illumination or distance, so they need a consistent surface of an object in different images) ignore the presence of specular pixels and work under the assumption of perfect diffuse surfaces. However, a vast majority of materials contain both diffuse and specular reflections in the real world. As a result, processing images with specular reflections using these algorithms can lead to significant inaccuracies [1, 16].

In recent years, various techniques try to handle the problem of specular reflections. Based on the number of input images, these methods could be divided into two main categories: multiple-image based and single-image based [1]. Multiple-image based approaches involve an image sequence of the same scene taken either from different viewpoints [16], with different illumination [17] or utilizing an additional polarizing filter. Nevertheless, obtaining such an image sequence is difficult, time-consuming or even impractical [18–23]. Single-image based approaches require color or texture analysis [23–25]. They achieve acceptable results in some images, but they are unstable when the analyzed image has complicated textures and extreme specularities [23].

Benefiting from computational photography [26] and light field (LF) imaging technologies [27, 28], we propose an accurate and novel framework that uses LF cameras to remove specularity. A handheld LF camera mounts an array of microlens in front of the sensor, which could record the full 4D rays to describe the scene, so one can refocus the image after a passive single–shot capture and shift viewpoints within sub-apertures of the main lens. With a LF camera to capture multiple views of the scene in a LF image, we could avoid the implementation complexity of conventional multiple-image based specular removal methods. In our algorithm, by exploiting the LF image to extract perspectives and modify focus, specular pixels could be detected and classified into “unsaturated” and “saturated” types, then replaced by their diffuse color via a color variance analysis of multiple views and a local color refinement. Thus, specular reflections could be substantially eliminated while the color consistency is well maintained in the rest part of the image. Moreover, the proposed algorithm can handle images which contain non-chromatic (i.e. R = G = B) and saturated specular pixels. It’s tested on various databases: the indoor and outdoor LF images taken by our Lytro ILLUM camera and the LF images from the Stanford light field archive [29]. It’s compared against three competitive methods: Tan et al. [24], Shen et al. [30] and Yang et al. [23].

The remainder of this paper is organized as follows. Section II introduces a brief review of the previous work related to highlight removal. Section III presents the basic knowledge of light field data, physical properties of reflection and dichromatic reflection model. In Section IV, we elaborate our algorithm in detail. We provide experimental results for real images in Section V. Lastly, concluded remarks are made in Section VI.

## Related work

### Multiple-image based highlight removal methods

This category utilizes a sequence of images, taking advantage of the different behaviors which these two reflections possess under specific conditions. Nayar et al. [31] achieved separation by incorporating polarization and color to obtain constraints on reflection components of each scene point, so the algorithm could work for textured surfaces. Unfortunately, obvious errors occur in the specular component on region boundaries due to chromatic aberration effects and mis-registration between polarization images. Later, Sato and Ikeuchi [32] examined a series of color images in a four-dimensional space and constructed a temporal-color space, which could describe the color change under the illumination densely varying with time. Lin and Shum [33] also changed the light direction to produce two color photometric images and estimated specular intensity from a linear model of surface reflectance, but when the surface color is similar to the illumination color, some specularity would be lost. In addition to that, light sources of the real world are usually fixed, especially in the outdoor scenes where light is not always controllable. These approaches have produced good results, but the need for polarization or changing light direction greatly restricts their applicability.

Consequently, a number of researchers tried to fix the illumination and vary viewpoints to make the decomposition. Their basic ideas mainly utilize the fact that when viewing from various directions, the color of diffuse reflection doesn’t change, but that of specular reflection or a mixture of the two does. Using multi-view color images, Lee and Bajcsy [34] proposed a spectral differencing algorithm to seek specularities. Later, Lin et al. [16] integrated this work with multi-baseline stereo to yield good separation; nevertheless, large baseline would lead to severe occlusions which might be mislabeled as specularity. Criminisi [35] looked into the Epipolar plane image (EPI) strips to detect specular pixels, but some artifacts showed up because of incorrect EPI-strip selection. Furthermore, configuring and adjusting the required cameramay not be easy.

### Single-image based highlight removal methods

In the last few years, considerable effort has been devoted to this category. For multi-colored images, many single-image based methods involve explicit color segmentation [36, 37] which is often non-robust for complex textures and specularities, or require user assistance for highlight detection [2]. Shafer [38], who introduced the dichromatic reflection model, proposed a method based on a simple knowledge: by spectral projection in color space, points on a single surface must lie within a parallelogram and be bounded by diffuse and specular colors. Klinker [37] classified color pixels as matte (diffuse reflection only), highlight (specular and diffuse reflections) and clipped (highlight that exceeds the camera dynamic range), then produced a skewed T shape color distribution. However, it may cause serious inaccuracies on textured surfaces whose distributions are not T shaped.

Avoiding segmentation, Tan and Ikeuchi [24] iteratively compared the intensity logarithmic differentiation of the input normalized image and the specular-free (SF) image to determine whether the normalized image contains only diffuse pixels. Shen et al. [30, 39] introduced a new modified SF image by adding a constant or pixel-dependent offset for each pixel. These SF image based methods can attain pleasing results on some images, but they require the input image has chromatic (R≠G≠B) surfaces because they heavily rely on color analysis [24]. They also need the specular component be pure white, or prior knowledge of illumination chromaticity which sometimes is not available. In addition, even the specular components are removed correctly, the original surface color may not be well preserved and produce dark diffuse images or noises. Yang et al. [23] proposed a real-time method by applying bilateral filtering to remove specularity. Although this method works robustly for many textured surfaces, it still cause artifacts at non-chromatic areas. Unlike many methods under an iterative framework, Nguyen et al. [40] provided a non-iterative solution by adopting tensor voting to get the reflectance distribution of an input image and removing specular and noise pixels as small tensors.

### Light field imaging

As an important branch of computational photography, light field imaging has been a fairly hot research topic in computer vision community, which offers new possibilities for many computer vision tasks. Modern LF rendering is firstly proposed by Levoy and Hanrahan [27] and Gortler et al.[28]. Early LF imaging systems use a camera array to capture the full scene, which is usually heavy and impractical for daily use. Ng [41] inserted a microlens array between the sensor and main lens, creating a portable plenoptic camera which enables consumers to conduct some basic post-capture applications, such as refocusing and altering viewpoints. Despite of its primary capabilities, LF cameras can be applied to various tasks, such as depth estimation [42, 43], saliency detection [44], matting [45] and super-resolution [46]. In this paper, we will explain how LF imaging is employed to specularity removal.

## Priori Conceptions

### Light field structure

**Fig 1:**
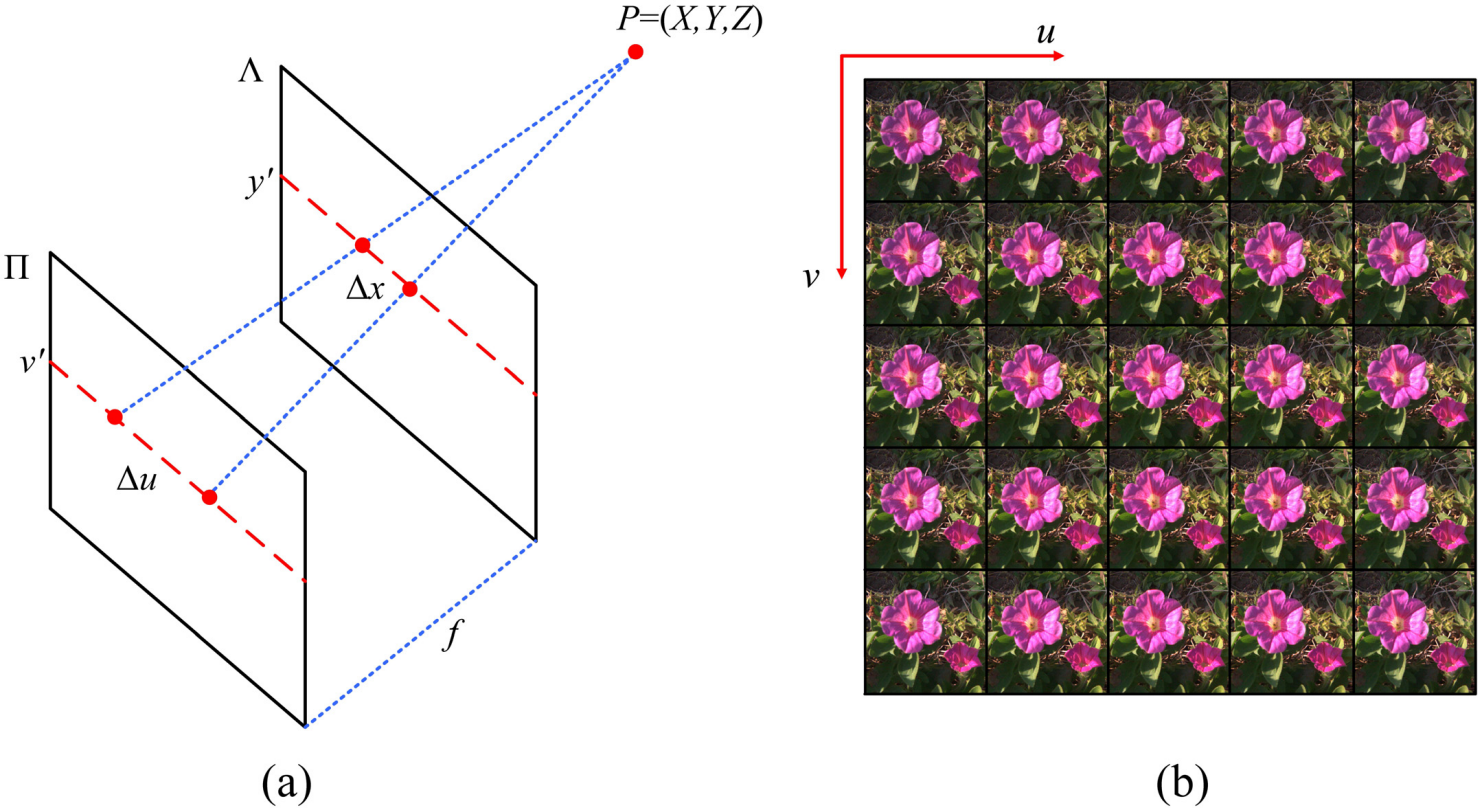
(a) Two-plane representation of a LF image. (b) The multiple pinhole images of a LF image after decoding. This LF image was taken by [43].

As noted in [27], a light field which is defined as radiance along rays in empty space is typically represented as a 4D function. A number of models have been proposed to describe light fields, such as the two-plane parameterization, sphere-sphere and sphere-plane parameterizations. In our paper, we represent the light field using the popular two-plane model, which records the intensity of a light ray passing through two parallel planes. For better understanding, it could be considered as a set of pinhole views from several viewpoints parallel to a common image plane in 3D space, as illustrated in Fig 1(a). The 2D plane ∏ contains the locations of viewpoints, which represents the angular domain and is parametrized by the coordinates (*u*,*v*), while the image plane Λ stands for the spatial domain and is parametrized by the coordinates (*x*,*y*). Hence, a 4D LF can be mapped by:

I:Λ×Π→ℝ,     (x,y,u,v)→I(x,y,u,v) (1)

By extracting the spatial pixels of the same viewpoints, we obtain multiple pinhole images where each represents an image captured from a slightly different perspective, as shown in Fig 1(b).

With a LF image, [41] clearly specifies how to achieve refocusing by shearing. Focusing at different depths is equivalent to changing the distance between the lens and the film plane, giving rise to a shearing of the light ray trace on the ray-space. By similar triangles and ray-space coordinates transforming, we can establish a 4D shear of the light field that enables refocusing at different depths below:

$$I_{\alpha }\left(x,y,u,v\right)=I_{0}\left(x+u\left(1-\frac{1}{\alpha }\right),y+v\left(1-\frac{1}{\alpha }\right),u,v\right)$$ (2)

where the shear value *α* is the depth ratio of the synthetic film plane to the actual film plane, *I*_0_ denotes the input LF image and *I*_*α*_ denotes the sheared LF image by *α*. In this paper, *α* is used to substitute depth of the scene for that they have a positive linear correlation with each other. Furthermore, when the light field is rearranged to focus at a scene point, all pixels of the same scene point from available views are obtained.

### Physical properties of reflection

To determine and separate two typical types of reflections: diffuse and specular, we first present a brief account of the formation process and the main differences between these two reflections. Theoretically, when light strikes an inhomogeneous opaque surface, it first passes through the interface between the air and the surface medium. Some light will promptly reflect back into the air producing specular reflection. The rest of light will penetrate through the body, undergo scattering from the colorant, and eventually be transmitted through the material, absorbed by the colorant, or re-emitted through the same interface by which it entered producing diffuse reflection. Therefore, the observed highlights on glossy surfaces are combinations of these two reflections.

Basically, there are three characteristic differences between diffuse and specular reflections (Table 1). First, they have different degrees of polarization (the percentage of the light being polarized), which is often used in separation methods involving polarization [31, 38]. Second, their intensity distributions follow different models, which are directly applied to describe and approximate these two components [24]. Third, for most inhomogeneous surfaces, the specular reflection takes the illumination color because it has the relative spectral power distribution (SPD) of the illuminant. In contrast, the color of the diffuse reflection is equal to the surface color since the SPD of the diffuse reflection is altered by the object’s body SPD (resulted from interactions with colorant particles) [37, 38]. This is the most common basis in reflection separation algorithms. Caused by the fundamental characteristics above, the two reflections also hold some other distinct properties, such as view-point dependence, color and geometric distribution, also shown in Table 1.

**Table 1:** Differences of diffuse and specular reflections.

	Polarization	Intensity Distribution Model	SPD	View-point Dependence	Color	Geometric Distribution
**Diffuse Reflection**	unpolarized	Lambert's Law	the object's body SPD	independent	the color of the object's surface	isotropic
**Specular Reflection**	highly polarized	the Torrance-Sparrow reflection model or the Beckmann-Spizzichino reflection model	the illumination's SPD	dependent	the color of the illumination	concentrated in a compact lobe

### Dichromatic reflection model

The dichromatic reflection model [38] is a simple reflectance model to determine the linear combinations of diffuse and specular reflection components from a standard color image. The total radiance *L* of inhomogeneous objects is the sum of two independent terms: the radiance *L*_*d*_ of the light reflected from the surface body and the radiance *L*_*s*_ of the light reflected at the interface:

$$L(\lambda ,l,v,n)=L_{d}(\lambda ,l,v,n)+L_{s}(\lambda ,l,v,n)$$ (3)

where *λ* is the light wavelength, *l* and *v* are the light source and camera viewpoint directions respectively, and *n* is the surface normal. Each component is decomposed into two parts:

$$L(\lambda ,l,v,n)=w_{d}(l,v,n)c_{d}(\lambda )+w_{s}(l,v,n)c_{s}(\lambda )$$ (4)

The magnitude term *w* is a geometric scale factor which only depends on geometry shapes, while the composition term *c* is a relative SPD which only depends on wavelength.

Note that the diffuse magnitude *w*_*d*_ only depends on *n* and *l*, whereas the specular magnitude *w*_*s*_ also changes with the camera viewpoint, resulting in the color intensity view angle dependent. For the sake of simplicity, we drop the *l* and *n* terms and project the scene by a digital camera. Therefore, a pixel *p* of the image is written as:

$$L(p)=w_{d}(p)c_{d}(p)+w_{s}(p)c_{s}(p)$$ (5)

where *B* and *G* indicate the color of the diffuse and specular reflection in the RGB channel.

## Proposed Method

**Fig 2:**
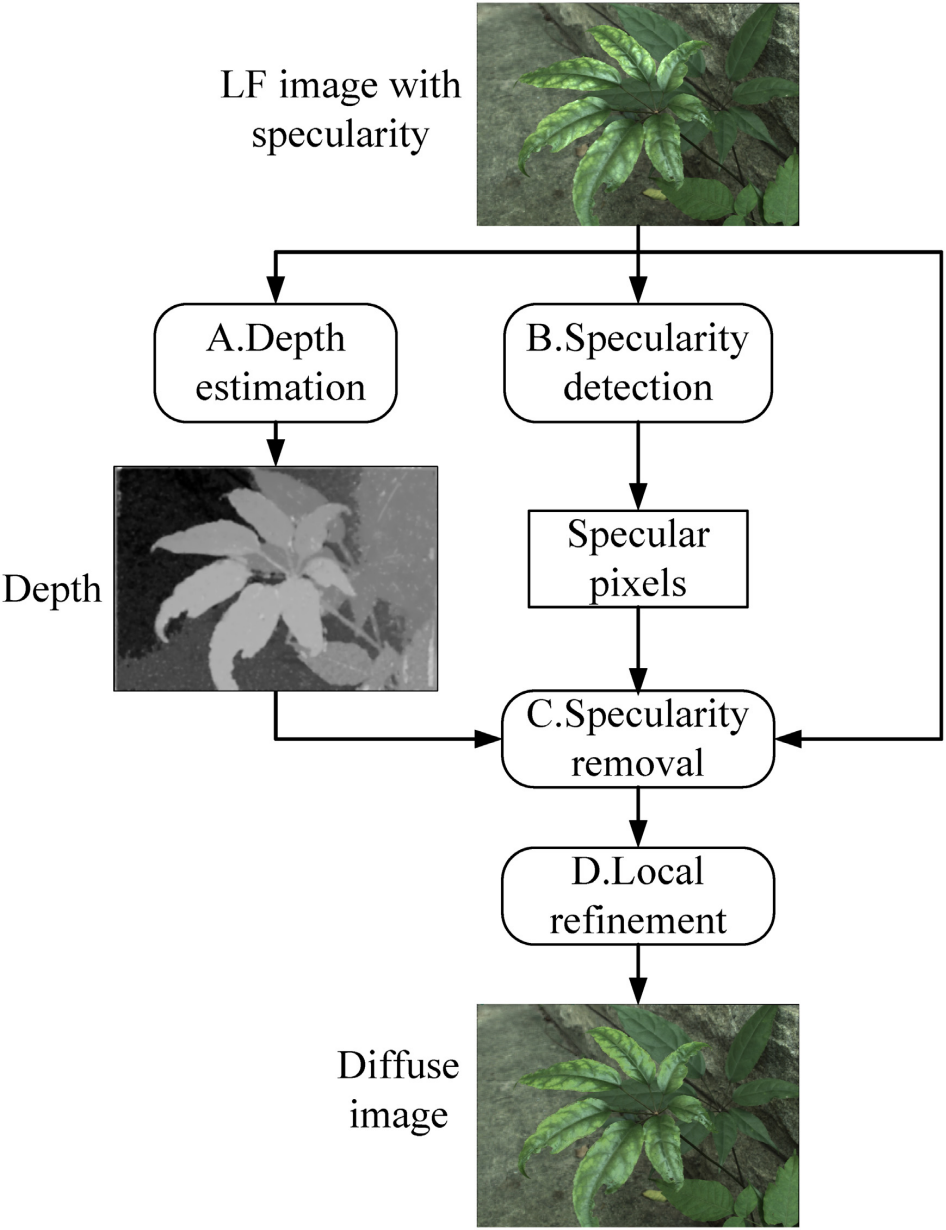
The framework of our algorithm.

As illustrated in Fig 2, our algorithm consists of four parts as depth estimation, specularity detection, specularity removal and local refinement.

### Light field image depth estimation

To achieve refocusing, a robust and accurate depth map of the LF image is required. Here we utilize the dense depth estimation algorithm by integrating both defocus and correspondence cues [42]. We firstly exploit the 4D epipolar image (EPI) derived from the LF data and make shears to operate refocusing. Then we present a simple contrast-based approach to compute the responses of two cues. With both local estimated cues, we combine them with a confidence measure and compute a global depth estimation using MRFs to get the final results.

Defocus cue: Depth from defocus has been actively investigated either through using several images exposures or a complicated device to capture the data in one exposure [47]. Employing a LF camera allows us to reduce the image acquisition requirements and record multiple angular information of the scene for estimating depth from defocus. Defocus measures the sharpness, or contrast within a patch. If a patch on a textured surface is refocused at the correct depth, it commonly provides the strongest contrast. A contrast-based measure is adopted here to find the optimal *α* with the highest contrast at each pixel. By taking the sheared EPI, the average value of pixel {*x*, *y*} is calculated:

$${\bar{I}}_{\alpha }\left(x,y\right)=\frac{1}{N_{\left(u,v\right)}}\sum_{\left(u\prime ,v\prime \right)}I_{\alpha }(x,y,u\prime ,v\prime )$$ (6)

where ${\bar{I}}_{\alpha }\left(x,y\right)$ is the sheared image, *N*_(*u*, *v*)_ is the number of angular pixels (*u*, *v*). By considering the spatial variance, the defocus cue is defined as:

$$D_{\alpha }(x,y)=\frac{1}{\left|W_{D}\right|}\sum_{(x\prime ,y\prime )\in W_{D}}\left|\Delta {\bar{I}}_{\alpha }\left(x\prime ,y\prime \right)\right|$$ (7)

where *W*_*D*_ is the window size of the current pixel and Δ is the spatial Laplacian operator using the full patch. Accordingly, we obtain a defocus response for every pixel in the image at each *α*.

Correspondence cue: LF cameras provide multiple views within the sub-apertures of the mainlens. Nevertheless, traditional multiple-view correspondence algorithms [16] require large baselines, so they are not suitable for short baseline settings of the LF camera [42]. Inspired by these traditional algorithms, we still aggregate the matching cost as correspondence cue over a window to estimate depth as well as allow for the characteristics of LF images. For a certain shear *α*, the matching cost for each spatial pixel is computed as the angular variance:

$$\sigma_{\alpha }(x,y)=\sqrt{\frac{1}{N_{\left(u,v\right)}}\sum_{\left(u\prime ,v\prime \right)}\left(I_{\alpha }(x,y,u\prime ,v\prime \right)-{\bar{I}}_{\alpha }(x,y){)}^{2}}$$ (8)

In an ideal case, if the matching cost for a spatial pixel *p* at α′ is zero, it means all the angular pixels corresponding to *p* stand for viewpoints that converge on a single point on the scene, so α′ corresponds to the optimal depth. However, due to noises, occlusions and specularities, it’s rather hard to find the ideal depth, so we search for the minima of the cost. For robustness, the variance is averaged in a small window *W*_*C*_:

$$C_{\alpha }(x,y)=\frac{1}{\left|W_{C}\right|}\sum_{(x\prime ,y\prime )\in W_{C}}\sigma_{\alpha }(x\prime ,y\prime )$$ (9)

For each pixel, both defocus and correspondence cues are obtained at different shear values. We maximize the spatial contrast for defocus and minimize the angular variance for correspondence across shears to find their optimal shear values $\alpha_{D}^{*}$ and $\alpha_{C}^{*}$:

$$\begin{array}{l} \alpha_{D}^{*}(x,y)=\begin{array}{c} \text{arg max}\\ \alpha \end{array}D_{\alpha }(x,y)\\ \alpha_{C}^{*}(x,y)=\begin{array}{c} \text{arg max}\\ \alpha \end{array}C_{\alpha }(x,y) \end{array}$$ (10)

Since the two cues may not reach their optimal values at the same *α*, their confidence are measured using Peak Ratio:

$$\begin{array}{l} D_{conf}(x,y)=D_{\alpha_{D}^{*}}(x,y)/D_{\alpha_{D}^{**}}(x,y)\\ C_{conf}(x,y)=C_{\alpha_{D}^{**}}(x,y)/C_{\alpha_{D}^{*}}(x,y) \end{array}$$ (11)

where α** is the next optimal *α* of defocus or correspondence cue. It produces higher confidence when the optimal *α* is significantly higher or lower than others, implying the estimation is more precise.

Defocus cue operates better at occlusions, repeating patterns and noise, so it produces consistent but blurry depth maps. Meanwhile, correspondence cue performs more robustly at bright or dark features of the image and preserves more defined depth results at edges, but is inconsistent in noisy regions. Fortunately, confidence measures enable us to combine the reliable region from each cue and acquire a globally optimized depth α*.

### Specularity detection

Specularity detection is essential to our algorithm. Specular pixels only account for a small percentage for most natural images, operating removal process on all pixels wastes massive time and storage. From observation, a glossy surface often exhibits color with higher intensities than a diffuse surface of the same color. In some extreme cases, the color and intensity of illumination dominate the appearance of highlights. If the light source color is uniform in every channel, the highlight pixels may tend to look white. Since many state-of-the-art specularity removal methods [24, 30] assume white illumination, they regard non-chromatic areas to be highlight and fail to separate specular and diffuse components in these areas.

**Fig 3:**
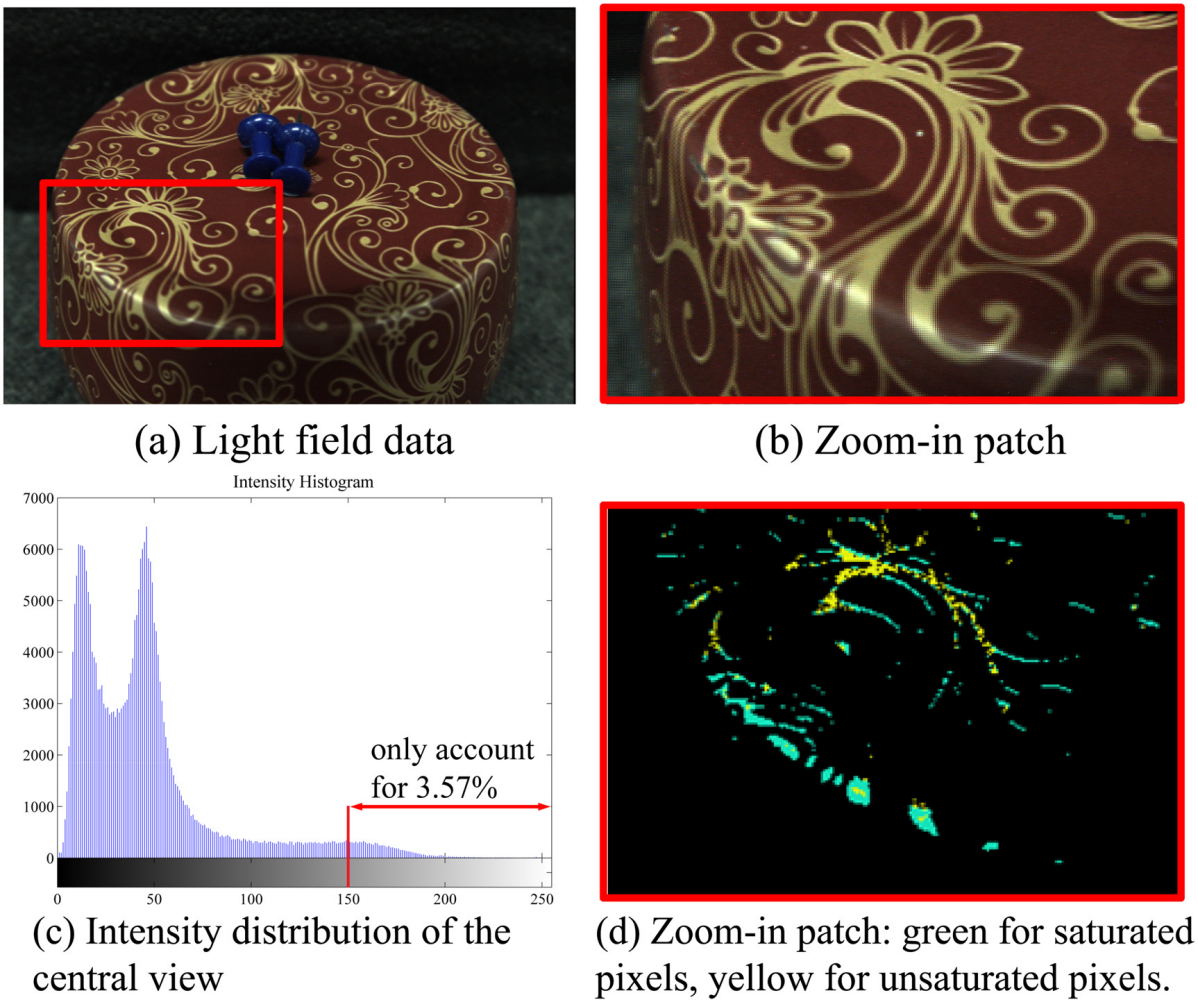
Specularity detection. (a) displays a LF image with the specular area (red patch) zoomed in (b). (c) shows specular candidates only take a tiny proportion of image. The detection results on the red patch are shown in (d).

As mentioned before, the color and intensity of highlight (specular) scene points differ largely when viewpoint changes. However, because the short baseline of LF cameras leads to a relatively smaller viewpoint change, points at strong or large highlight areas may change slightly in color and intensity. We propose a simple threshold strategy to efficiently detect and classify specular points into “unsaturated” and “saturated” types: a saturated scene point displays highlight in all (*u*, *v*) views, while an unsaturated point presents various combinations of diffuse and specular color in different views. This strategy works like this: In the central-view image, a pixel *p* whose intensity is higher than a given threshold *h*_*thres*_ is labelled as “specular candidate”. *h*_*thres*_ can be adjusted within [0,255] according to the lowest intensity of specular pixels. Then, the pixels of the same candidate under all views are located by refocusing to its estimated depth and their variance are assessed. If the variance exceeds a given threshold *var*_*thres*_, *p* is accepted as “unsaturated”. Otherwise, *p* is “saturated” or it reflects non-chromatic diffuse color. In implementation, we set 0.002 for *var*_*thres*_ and 150 for *h*_*thres*_. Fig 3 shows an example of specularity detection.

### Specularity removal

This section deals with “unsaturated” specular pixels to recover their original diffuse color. The depth map that was generated before is applied to refocus and create multiple views. For an “unsaturated” pixel {*x*, *y*} in the central view, we remap the original LF image *I*_0_ at its depth α*(*x*, *y*) to obtain the same scene points in all (*u*, *v*) views according to Eq 1. Then, conduct color analysis within *u*, *v* of each *x*, *y*: we use k-means clustering to classify them into two clusters in HSI color space and record their centroids. We denote the cluster whose centroid has a higher intensity as *diffuse+specular* set with the centroid color *M*_1_, and the other cluster as *diffuse only* set with the centroid color *M*_2_. Based on the dichromatic reflection model, if the magnitude *w*_*d*_ and *w*_*s*_ are set to 1, the two centroids are written as:

$$\begin{array}{l} M_{1}=B+G\\ M_{2}=B \end{array}$$ (12)

where *B* and *G* represent the color of diffuse and specular components. With known *M*_1_ and *M*_2_, *G* could be determined by simply subtracting the two equations.

We also offer a confidence metric to measure the accuracy for each unsaturated pixel. Assuming that higher confidence occurs at the pixel with higher *M*_1_ intensity and larger two-centroid distance, the metric is constructed as:

$$conf=\text{exp}(-\frac{\beta_{0}}{\left|M_{1}\right|}-\frac{\beta_{1}}{\left|M_{1}-M_{2}\right|}+\frac{\beta_{2}}{R})$$ (13)

where *R* is the average intra-cluster distance, *β*_0_, *β*_1_, *β*_2_ are constant parameters. In our implementation, both *β*_0,_ *β*_1_ are set to 0.5, *β*_2_ is set to 1.

For each pixel labeled as “unsaturated”, we subtract the specular term *G* to restore their original diffuse color. By looking through a small window around *x*, *y*, *u*, *v*, we compute a weighted *G* by favouring higher confidence and smaller difference between *I*_0_(*x*, *y*, *u*, *v*) and its neighbor’s *M*_1_. The specular component is removed by:

$$\begin{array}{l} I_{d}\left(x,y,u,v\right)=I\left(x,y,u,v\right)-\langle w\times \left|M_{1}\left(x^{\prime },y^{\prime }\right)-M_{2}\left(x^{\prime },y^{\prime }\right)\right|\rangle \\ w=\text{exp}\left\{-\gamma /\left(Conf\left(x^{\prime },y^{\prime }\right)\times \left|I\left(x,y,u,v\right)-M_{1}\left(x^{\prime },y^{\prime }\right)\right|\right)\right\} \end{array}$$ (14)

where *x*′,*y*′ are within the search window around *x*, *y*, *u*, *v*, and 〈.〉 represents the expected value. We use a 15 × 15 window and 1 for *γ* in implementation.

### Local refinement

Due to the small baseline, a scene point at specular regions which is saturated in all viewpoints is common. Angularly saturated pixels exhibit the strongest intensity of the light source color and totally lose their diffuse terms. Removing specularities in the former step only takes effect on unsaturated pixels, so it may create highlight holes in the middle of the specular area where saturated pixels always occur. To remove specularities entirely, we apply a local color refinement to fill these holes and gain a final diffuse image. We assume that the color and texture of a point vary smoothly in its local area, which holds true for most specular images. Consequently, the color information of saturated pixels could be remedied with the color information of its neighbors.

We implement this by using K nearest neighbors. For a particular saturated pixel, we find k nearest points that are non-specular around it and assign weight to constrain the nearer neighbors contribute more to the average than more distant ones. Then, the corresponding pixels of the saturated point {*x*, *y*} in all (*u*, *v*) views are replaced by averaging its neighbors {*x*^*i*^, *y*^*i*^ |*i* = 1, … *k*}.

$$I_{r}(x,y,u,v)=\{\begin{array}{l} \sum_{i=1,\ldots k}\frac{1}{w_{i}}I_{0}(x^{i},y^{i},u,v),\text{ if}\{x,y\}\text{ is saturated},\\ I_{0}(x,y,u,v),\text{ others}. \end{array}$$ (15)

where *w*_*i*_ = 2^*i*^. In our study, k is set to 4 to achieve steady and reliable results.

## Experiments & Comparisons

To validate the effectiveness of our proposed approach, we test it on multiple images with multi-color and highly textured surfaces captured by Lytro ILLUM, together with the LF images which are captured by a commercial Canon Digital camera fixed on a moving Lego Mindstorms gantry from the Stanford light field archive. For Lytro ILLUM, indoor scenarios are taken under controlled illumination condition (incandescents) and outdoor scenarios are under uncontrolled wild environment (sunlight). The camera parameters: exposure: ISO: auto, focal length: 9.5–77.8 mm (30–250 mm equivalent), lens aperture: Constant *f*/2.0. Considering views on the borders of the main lens do not capture light as much as the views on the center, only the central 7 × 7 views are used to construct the LF image.

We compare our work against three currently popular single-image based algorithms: Tan et al. [24], Shen et al. 30] and Yang et al. [23]. Their source code is freely available on the authors’ websites [48–50]. We only make comparisons with the single-image based algorithms because the existing multiple-image based techniques require images taken under various conditions or have large baselines, making it impracticable to compare with them in the same setting. To acquire a single image input, the original LF data is refocused to the specular area. Then the diffuse output is generated under the authors’ default settings. We also refocus our refined LF image at the same depth for comparison.

### Qualitative analysis

#### Recovering the LF diffuse image

**Fig 4:**
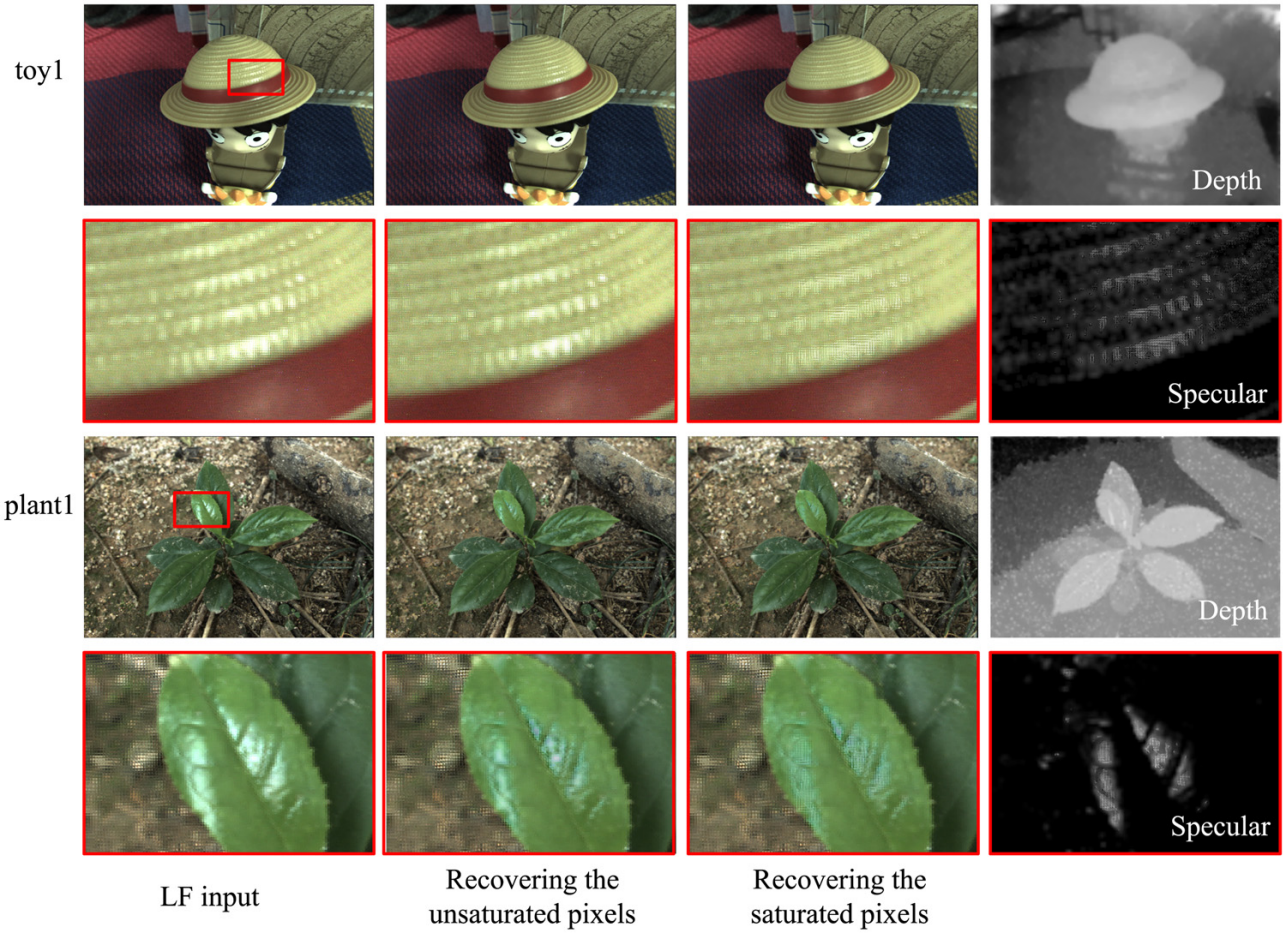
Two processing examples of the proposed method. The first and third rows (from left to right) display the LF input image, LF image after recovering unsaturated pixels, LF image after recovering saturated pixels, and the estimated depth of the central view. The specular area is marked by a red patch. The second and fourth rows show the zoom-in specular areas of the upper corresponding LF images, and specular components.

Fig 4 illustrates one indoor and one outdoor example of our proposed method. The displayed images are the 4D LF images which contains cropped microlens-images, except that the depth map is from the central pinhole image. By zooming in the specular area on the hat and the leaf, we easily observe that unsaturated and saturated pixels are correctly restored to their original diffuse color in two steps. The close-up patch of specular component is also provided. Note that the color of specular components have been enhanced for easier visibility throughout this paper.

#### Diffuse results for Lytro ILLUM images

**Fig 5:**
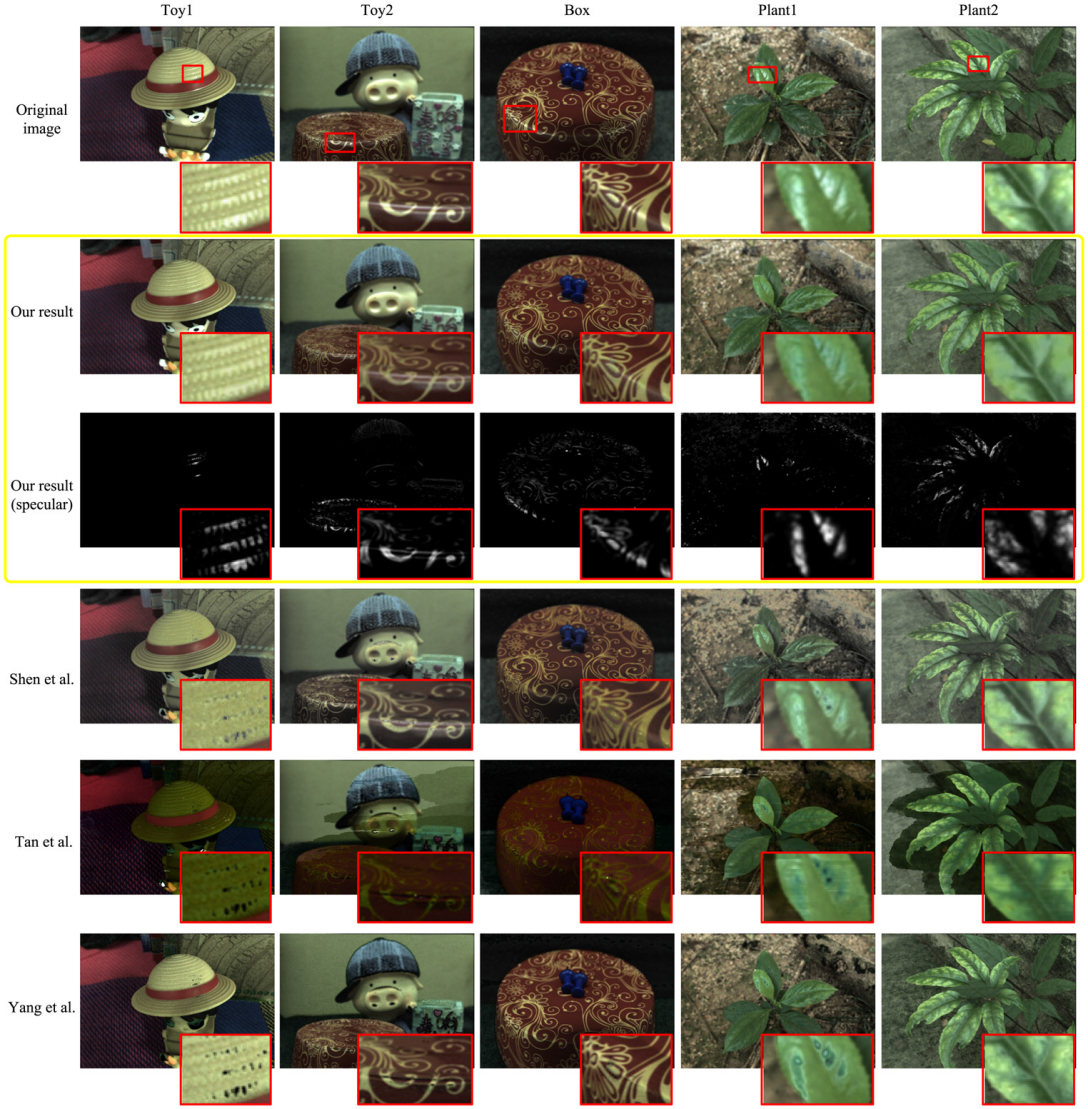
Diffuse results for Lytro ILLUM images. The first row shows the refocused original images and their corresponding zoom-in specular areas. The second and third rows are diffuse and specular images of our proposed method. The other three rows are the diffuse images of Shen et.al. [30], Tan et.al. [24] and Yang et.al. [23].

In Fig 5, the indoor and outdoor objects have glossy surfaces which are marked by red rectangles. Our approach correctly weakens the specular intensity, successfully recovers diffuse color, and well preserves the consistency of other regions, while Shen, Tan and Yang can cause obvious mistakes, particularly at white and textured areas. In detail, Shen’s method creates black holes on the non-chromatic area (the eyes in Toy1 and nose in Toy2) and the highlight in Plant1 is not removed completely yet. Besides, the color of diffuse areas slightly differs to the original image (the hat in Toy1, the face in Toy2). Anyway, it produces good results on five images. About the color information, Tan’s results are significantly darker than the original images and bring easy-to-see errors, e.g. losing color consistency, losing texture information and creating inexistent edges. Yang produces comparative results in Plant2 and Box, but gives rise to black holes in Toy1 and Toy2 and inconsistent color in Plant1. Non-specular areas have been ruined to different degrees in these methods, which largely reduce image quality. The results show our algorithm outperforms them.

#### Diffuse results for Stanford light field archive images

**Fig 6:**
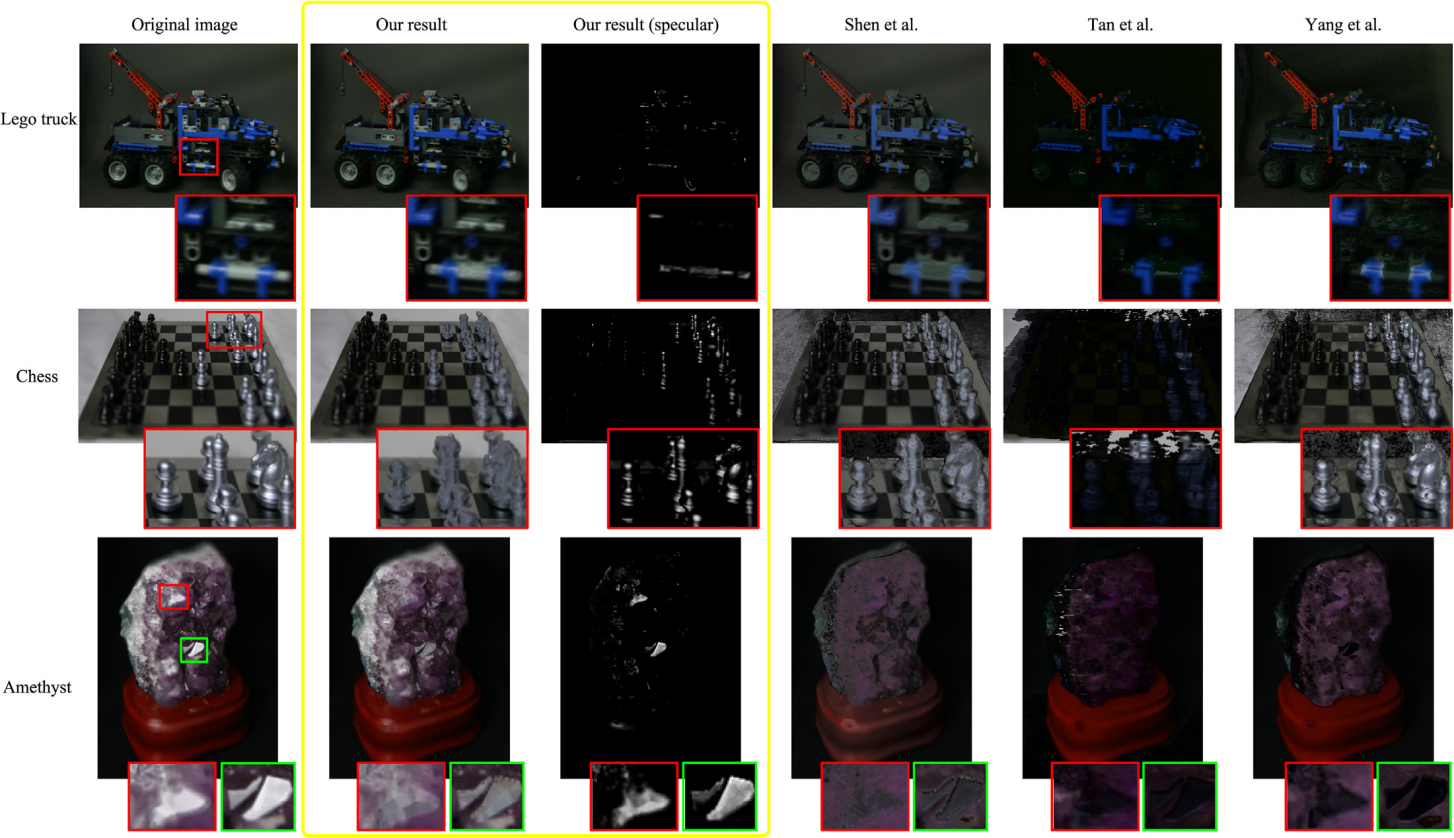
Experiments of the Stanford light field archive.

Fig 6 illustrates three challenging images from the Stanford light field archive. The first image is a Lego Technic truck which has very complex geometry. Our proposed method properly reduces the highlight on the wheels without damaging its geometry and brightness. The second image is a chess board with pieces, which have specular reflections of various intensities. Compared to our diffuse result, Tan loses almost all the information of the image while Shen and Yang destroy the color constancy of background. The last is a chunk of amethyst with interesting specularities and some translucency. After the processing steps for unsaturated and saturated pixels, strong highlights in red and green rectangles are removed acceptably. However, the rest methods fail at it.

### Quantitative analysis

#### Subjective evaluation metrics

To evaluate our approach in a quantized way, we invite 50 volunteers from different gender and age to score the specular removal results of all methods. Given an image with specular items, human’s brain can automatically fix the specular parts and illustrate the diffuse version of this image. Since it’s rather individual dependent, for each image, we omit the highest/lowest score and average scores to decrease subjective bias. For every image, the volunteers are required to rate two indexes: SA and IQ. SA is for specularity accuracy, which is graded based on the accuracy of specular components separated by each method compared with the one he has in mind. IN is for image naturalness because preserving naturalness is essential for highlight removal methods to achieve pleasing perceptual quality. Both the two scores are ranging from 0~100, where 0~20 means very poor, 20~40 means poor, 40~60 means fair, 60~80 means good and 80–100 means very good. The greater value of the score, the better quality of the image. The average scores are shown in Table 2, clearly our algorithm is superior to the other three methods in most specular images.

**Table 2:** The scores of the specular removal results.

	Toy1		Toy2		Box		Plant1		Plant2		Lego truck		Chess		Amethyst	
	SA	IN	SA	IN	SA	IN	SA	IN	SA	IN	SA	IN	SA	IN	SA	IN
**Proposed method**	**80.1**	**85.3**	**82.1**	**87.1**	82.2	79.6	**85.5**	**89.6**	**88.7**	**90.7**	**69.0**	**78.3**	**81.4**	**74.4**	**82.5**	**80.1**
**Shen et al.**	68.4	72.5	60.7	69.5	83.4	75.4	57.8	66.2	81.4	85.2	64.5	69.2	77.4	51.9	47.8	59.4
**Tan et al.**	53.2	45.0	47.5	37.5	59.8	51.2	55.2	40.1	50.6	47.5	35.3	32.7	15.3	10.3	27.2	20.4
**Yang et al.**	73.4	69.3	65.2	59.8	**86.7**	**82.3**	72.3	69.6	67.4	77.0	60.2	59.4	33.0	36.6	56.0	63.3

#### Objective evaluation metrics

The volunteers are also asked to mark the areas where highlights occur. We average the manually marked regions and regard them as the highlight area ground truth to provide an objective evaluation. Note that this manually marked regions doesn’t contain the highlight intensity information, so we change the specular component image of each scene to a binary image under the control of the same threshold. Then the basic measures used in evaluating classification results: precision, recall and F-measure (harmonic mean of precision and recall compared to ground truth) are calculated to test the accuracy of highlight detection, as shown in Tables 3, 4 and 5. From our observation, people tend to draw lines around the relatively stronger and larger highlight areas, and overlook weak and small ones. Our algorithm has a higher precision than other methods among most images while the recall of ours is actually common. The F-measure, which take the performance of both precision and recall into consideration, demonstrates the effectiveness of our method. The precision of other techniques is much lower because non-chromatic (white or gray) areas which they regard as highlights are possibly not included in the manually marked highlights. In addition, Tan achieves a considerably high recall, but it suffers from a poor precision, because it includes most true highlight pixels at the expense of even more detected false highlights.

**Table 3:** The precision of the specular removal results.

	Toy1	Toy2	Box	Plant1	Plant2	Lego truck	Chess	Amethyst
**Proposed method**	**0.9634**	**0.8570**	0.8121	**0.8681**	0.7862	**0.3152**	**0.7137**	**0.8311**
**Shen et al.**	0.0548	0.7198	**0.9952**	0.0381	**0.8293**	0.1257	0.0230	0.0664
**Tan et al.**	0.0402	0.0823	0.4067	0.0073	0.0425	0.0528	0.0496	0.0759
**Yang et al.**	0.0656	0.2488	0.8832	0.0303	0.0691	0.0643	0.0263	0.0699

**Table 4:** The recall of the specular removal results.

	Toy1	Toy2	Box	Plant1	Plant2	Lego truck	Chess	Amethyst
**Proposed method**	0.5313	0.2024	0.3121	**0.3379**	0.3274	0.3042	0.6522	0.5155
**Shen et al.**	0.1573	0.2625	0.2046	0.0070	0.1173	0.4857	0.3780	0.4809
**Tan et al.**	**0.9676**	**0.7911**	**0.3734**	0.1103	**0.5158**	**0.7771**	**0.9873**	**0.6572**
**Yang et al.**	0.2940	0.4354	0.2643	0.0752	0.1914	0.5139	0.2364	0.6336

**Table 5:** The F-measure of the specular removal results.

	Toy1	Toy2	Box	Plant1	Plant2	Lego truck	Chess	Amethyst
**Proposed method**	**0.6849**	0.3275	**0.4509**	**0.4865**	**0.4623**	**0.3096**	**0.6816**	**0.6363**
**Shen et al.**	0.0813	**0.3847**	0.3394	0.0118	0.2055	0.1997	0.0434	0.1167
**Tan et al.**	0.0772	0.1491	0.3893	0.0137	0.0785	0.0989	0.0945	0.1361
**Yang et al.**	0.1073	0.3167	0.4068	0.0432	0.1015	0.1143	0.0473	0.1259

## Conclusions

We have presented a novel and accurate specular reflection removal methodology based on light field imaging, which extensively exploits the spatial and angular information of the 4D LF together with the characteristics of diffuse and specular reflections. In our methodology, the diffuse image is first recovered by removing the specular effects on “unsaturated” pixels, and then refined locally on “saturated” pixels. The classification and recovering steps on two types of specular pixels makes our approach applicable to various surfaces with more complex and stronger highlights. We have experimented on multiple real world LF images from our Lytro ILLUM and the Stanford light field archive with both qualitative and quantitative analysis. The results demonstrate that our algorithm achieve excellent performance, especially in non-chromatic and textured areas, while properly preserving color constancy on non-specular areas. Still, our method does not work well at mirrors or extremely specular surfaces. Achieving higher accuracy of specularity detection and improving robustness of a larger area of highlight are left as future works.
